# Epidemiology and molecular characteristics of respiratory syncytial virus (RSV) among italian community-dwelling adults, 2021/22 season

**DOI:** 10.1186/s12879-023-08100-7

**Published:** 2023-03-07

**Authors:** Donatella Panatto, Alexander Domnich, Piero Luigi Lai, Matilde Ogliastro, Bianca Bruzzone, Cristina Galli, Federica Stefanelli, Elena Pariani, Andrea Orsi, Giancarlo Icardi

**Affiliations:** 1 Department of Health Sciences, University of Genoa, Via A. Pastore, 1, 16132 Genoa, Italy; 2 Interuniversity Research Center on Influenza and Other Transmissible Infections (CIRI-IT), Genoa, Italy; 3 Hygiene Unit, San Martino Policlinico Hospital-IRCCS for Oncology and Neurosciences, Genoa, Italy; 4 Department of Biomedical Sciences for Health, University of Milan, Milan, Italy

**Keywords:** Respiratory syncytial virus, RSV, Adult population, RSV epidemiology, RSV molecular characteristics

## Abstract

**Background:**

Respiratory syncytial virus (RSV) is a leading cause of acute respiratory infections worldwide. While historically RSV research has been focused on children, data on RSV infection in adults are limited. The goal of this study was to establish the prevalence of RSV in community-dwelling Italian adults and analyze its genetic variability during the 2021/22 winter season.

**Methods:**

In this cross-sectional study, a random sample of naso-/oropharyngeal specimens from symptomatic adults seeking for SARS-CoV-2 molecular testing between December 2021 and March 2022 were tested for RSV and other respiratory pathogens by means of reverse-transcription polymerase chain reaction. RSV-positive samples were further molecularly characterized by sequence analysis.

**Results:**

Of 1,213 samples tested, 1.6% (95% CI: 0.9–2.4%) were positive for RSV and subgroups A (44.4%) and B (55.6%) were identified in similar proportions. The epidemic peak occurred in December 2021, when the RSV prevalence was as high as 4.6% (95% CI: 2.2–8.3%). The prevalence of RSV detection was similar (*p* = 0.64) to that of influenza virus (1.9%). All RSV A and B strains belonged to the ON1 and BA genotypes, respectively. Most (72.2%) RSV-positive samples were also positive for other pathogens being SARS-CoV-2, *Streptococcus pneumoniae* and rhinovirus the most frequent. RSV load was significantly higher among mono-detections than co-detections.

**Conclusion:**

During the 2021/22 winter season, characterized by the predominant circulation of SARS-CoV-2 and some non-pharmaceutical containment measures still in place, a substantial proportion of Italian adults tested positive for genetically diversified strains of both RSV subtypes. In view of the upcoming registration of vaccines, establishment of the National RSV surveillance system is urgently needed.

## Background

Human respiratory syncytial virus (RSV) is a leading cause of acute respiratory infections (ARIs) worldwide and belongs to the *Paramyxoviridae* family, genus *Orthopneumovirus* [1]. Of 11 proteins encoded by the viral RNA, glycoproteins G and F play the prominent role in the virus–host interaction: the latter functions as fusion agent, while the former is responsible for cell attachment [2]. The F protein is highly conserved and immunogenic, which makes it particularly attractive for vaccine development. By contrast, the G protein is subjected to frequent mutations and, on the basis of this latter, RSV is classified into two antigenically distinct subgroups A (RSV-A) and B (RSV-B). In a typical season, both subgroups co-circulate, although one usually predominates over the other [3–5].

Traditionally, research on the epidemiology and burden of RSV infections has been focused on young children. Indeed, RSV was first isolated in a child with bronchopneumonia [6], and RSV is responsible for a large burden of disease in infants [7–9], typically exceeding that of seasonal influenza [10]. However, the burden of disease in adults is considerable as well. For instance, it has been estimated that in 2015 in industrialized countries a total of 1,500,000 (95% CI: 300,000–6,900,000) episodes of RSV-associated ARIs occurred among older adults and 14.5% of these patients required hospitalization [11].

Since the early 2020, non-pharmaceutical interventions adopted to limit the spread of severe acute respiratory syndrome coronavirus 2 (SARS-CoV-2) have modified seasonal circulation pattern of other respiratory viruses, including RSV. Most available surveys [12–14] have reported a dramatic decrease in RSV detections during the different waves of COVID-19 pandemic. On the other hand, in Shenzhen (China) contrary to seasonal influenza, the detection rate of RSV in children < 14 years increased from 6.6% in 2019 to 25.6% in 2020 [15]. RSV and SARS-CoV-2 co-occurrence seems to be infrequent: a systematic review and meta-analysis by Musuuza et al. [16] has estimated the prevalence of SARS-CoV-2 and RSV co-infection to be 3.8%, and RSV superinfection in SARS-CoV-2-positive individuals to be 0.4%. A recent large study [17] conducted among hospitalized SARS-CoV-2-positive adults in the United Kingdom has revealed a prevalence of RSV of 3.16%.

To date, there is no centralized surveillance system of RSV in Italy and previous research has been mostly focused on the pediatric population [18]. Few available epidemiological surveys have demonstrated a substantial RSV burden in the Italian adults. For instance, in four consecutive winter seasons (from 2014/15 to 2017/18) a total of 3.3–12.2% and 8.1–14.2% of influenza-like illness (ILI) cases in working-age and older adults, respectively, were caused by RSV [19]. During the 2019/20 season, 22.6% of hospitalized Italian adults with respiratory symptoms were diagnosed with RSV [20]. With regard to RSV viral population, a gradual increase in detections of the RSV-A genotype ON1 has been reported, while RSV-B is currently represented by BA strains [19, 21, 22]. Similarly to other countries, during the first COVID-19 pandemic waves, circulation of RSV in Italy decreased significantly [23–25]. However, a sharp increase in RSV detections among young children was observed in late 2021 [26], suggesting resurgence of RSV in Italy [27]. To our knowledge, no studies on the epidemiology of RSV infection in adults have been conducted in Italy since the start of COVID-19 pandemic. The objective of this study was to quantify the prevalence of RSV (co)-detections and analyze its genetic variability among symptomatic community-dwelling adults seeking for SARS-CoV-2 molecular diagnosis during the 2021/22 winter season.

## Methods

### Study population

In this cross-sectional study, nasopharyngeal and/or oropharyngeal swab specimens from symptomatic community-dwelling adult individuals seeking for SARS-CoV-2 molecular diagnostics were tested for RSV and other respiratory pathogens. In particular, eligible specimens came from subjects self-presenting at a community point for SARS-CoV-2 swab with any respiratory symptom(s). These samples were collected between December 1, 2021 and March 31, 2022 and processed at the regional reference laboratory for COVID-19 diagnostics located at San Martino Polyclinic Hospital (Genoa, Italy). The study period was characterized by the highest SARS-CoV-2 incidence [28], which was driven by the Omicron variant of concern [29]. Individuals of both sexes, aged ≥ 18 years and residing in the Metropolitan City of Genoa were eligible.

The minimum sample size was determined *a priori* as follows: by assuming a true RSV prevalence of 3% when *α* is 95% and precision is 0.01, at least 1,118 samples were required. By assuming that 15% of specimens would have an insufficient volume, a total of 1,286 samples eluted in the universal transport medium (UTM) (Copan Italia S.p.A.; Brescia, Italy) were randomly extracted from the available set of specimens stored at -80 °C.

### Molecular detection of RSV by real-time RT-PCR

Each specimen was first screened for RSV by means of the Resp-4-Plex kit (Abbott Molecular Inc., Des Plaines, IL, USA) used with the fully automated Alinity m System (Abbott Molecular Inc., Des Plaines, IL, USA) and according to the manufacturer’s instructions. This kit is a multiplex real-time reverse transcription polymerase chain reaction (RT-PCR) for the qualitative detection and differentiation of RNA from SARS-CoV-2, RSV, influenza A and B viruses. According to the manufacturer, the limit of detection is 0.300 and 0.100 median tissue culture infectious dose (TCID_50_)/ml for RSV-A and RSV-B, respectively [30].

To discern RSV subgroup and detect the presence of other respiratory pathogens, samples positive for RSV were further tested by means of the Allplex Respiratory Panel (RP) assays (Seegene Inc.; Seoul, Republic of Korea) according to the manufacturer’s instructions. Briefly, nucleic acids were first extracted using the STARMag Universal Cartridge Kit (Seegene Inc.; Seoul, Republic of Korea) on the automated Nimbus IVD (Seegene Inc.; Seoul, Republic of Korea) platform. For this purpose, 200 *µ*l of each specimen was extracted and eluted with 100 *µ*l of elution buffer and set up for RT-PCR. RT-PCR was then performed on a CFX96 instrument (Bio-Rad Laboratories, Inc; Hercules, CA, USA) with the Allplex RPs 1–4 kits. These four panels are able to detect the most common respiratory pathogens - both viruses [RP 1: RSV-A, RSV-B, influenza viruses A, A(H1N1), A(H1N1)pdm09, A(H3N2) and B; RP 2: adenovirus (AdV), enterovirus (EV), metapneumovirus (MPV), parainfluenza (PIV) viruses 1–4; RP 3: bocaviruses (BoV) 1–4, coronaviruses (CoV) 229E, NL63, OC43, rhinovirus (RV)] and bacteria [RP 4: *Streptococcus pneumoniae* (SP), *Bordetella parapertussis* (BPP), *Bordetella pertussis* (BP), *Chlamydophila pneumoniae* (CP), *Haemophilus influenzae* (HI), *Legionella pneumophila* (LP), *Mycoplasma pneumoniae* (MP)]. For each RT-PCR, 8 *µ*l of the extracted nucleic acid in a final volume of 25 *µ*l was used. The diagnostic accuracy of this assay in detecting RSV-A and RSV-B is 100% [31].

Samples showing cycle threshold (Ct) values < 40 in at least one assay were deemed positive. Ct values were used as a proxy measure of viral load: lower Ct values indicate higher viral load.

### Sequencing and molecular characterization of RSV A and B

All samples tested positive in either commercial RT-PCR assay were sequenced. Viral RNA was first extracted from 140 *µ*l of sample using the QIAamp Viral RNA Mini Kit (QIAGEN; Hilden, Germany) and eluted in 50 *µ*l of the supplied elution buffer. RT-PCR on the extracted RNA was then performed using the SuperScript IV One-Step RT-PCR System (Invitrogen; Carlsbad, CA, USA) with the following temperature profile: 1 cycle at 45 °C for 30 min and 98 °C for 2 min, 40 cycles at 98 °C for 30 s, 55 °C for 30 s, 72 °C for 2 min and a final extension of 72 °C for 5 min.

The first amplification of gene G (irrespective of the RSV subgroup) was performed by using specific primers [32]. Subsequently, a nested PCR using 5 *µ*l of the amplified cDNA as template was performed. The amplification was carried out using Platinum II Taq Hot-Start DNA Polymerase (Invitrogen; Carlsbad, CA, USA) with the following conditions: 1 cycle at 94 °C for 2 min, 40 cycles at 94 °C for 30 s, 55 °C for 30 s, 68 °C for 2 min and a final extension at 68 °C for 5 min.

Full G gene amplicons of 969 bp and 954 bp of RSV-A and RSV-B, respectively, were visualized on 1.5% agarose gel with Midori Green Direct (NIPPON Genetics EUROPE; Düren, Germany) as intercalating dye. PCR products were then purified with ExoSAP-IT PCR Product Cleanup Reagent (Applied Biosystems; Waltham, MA, USA) as per manufacturer’s instructions. Finally, the sequencing reactions were performed on SeqStudio Genetic Analyzer System (Applied Biosystems; Waltham, MA, USA) for Sanger sequencing. The obtained sequences were assembled with SeqScape Software v4.0 (Applied Biosystems; Waltham, MA, USA).

### Phylogenetic analysis of RSV-A and RSV-B

Overlapping sequences of the G gene were edited and assembled by using the ClustalW program implemented in the BioEdit software v. 7.2 [33]. All nucleotide sequences obtained were deposited in GenBank database (https://www.ncbi.nlm.nih.gov/genbank/) under the accession numbers (ANs) ON707106–ON707123. Multiple sequence alignments, including study and reference sequences, were carried out by using the ClustalW program, implemented in the BioEdit software. To identify RSV-A and RSV-B genotypes, nucleotide sequence alignments were phylogenetically analyzed by MEGA software, version 6.0 [34].

Nucleotide sequence identities were established by generating sequence identity matrices implemented in BioEdit and expressed as mean percentage with ranges. Cluster analysis of the study sequences was conducted in MEGA. Phylogenetic trees were generated by using the maximum likelihood method based on the Hasegawa-Kishino-Yano and the Tamura-Nei model with a gamma-shaped distribution model for RSV-A and RSV-B, respectively. The best amino acid substitution models were ascertained by analyzing sequence datasets within the “Models tool” implemented in MEGA. Confidence estimates were assessed by conducting a bootstrap analysis with 1,000 replicates and estimates ≥ 70% were considered significant. The phylogenetic relationship among study strains was assessed by calculating pairwise p-distances expressed as averages crude rates ± standard deviations (SDs).

### Statistical analysis

Prevalence was expressed as percentage with Clopper-Pearson exact 95% confidence intervals (95% CIs). The McNemar’s test was used to compare the overall prevalence of RSV and influenza virus detections. Prevalence estimates were compared by means of the Fisher’s exact test. Unpaired *t* test was used to compare Ct values between RSV mono-detections and co-detections and the corresponding effect size was expressed as Cohen’s *d*. The Firth’s penalized logistic regression was applied to estimate the adjusted (for age, sex and month of swab) odds ratio (aOR) of testing positive for RSV according to the SARS-CoV-2 positivity status.

Statistical analysis was performed using R stat packages v. 4.1.0 (R Core Team; Vienna, Austria) [35].

## Results

### Prevalence of RSV

A total of 1,213 samples were tested by the Resp-4-Plex assay and the main characteristics of subjects are reported in Table 1.

**Table 1:** Characteristics of adults with respiratory symptoms included in this study and results of SARS-CoV-2 RNA detection in their naso- or oropharyngeal samples (*n* = 1,213)

Variable	Level	% (*n*)	95% CI
Sex	Female	58.3 (707)	55.5–61.1
	Male	41.7 (506)	38.9–44.5
Age, years	18–64	53.1 (644)	50.2–55.9
	≥ 65	46.9 (569)	44.1–49.8
	Median	61	43–80^a^
Month of sample	Dec 2021	18.0 (218)	15.8–20.3
	Jan 2022	29.3 (355)	26.7–31.9
	Feb 2022	31.7 (384)	29.0–34.4
	Mar 2022	21.1 (256)	18.8–23.5
SARS-CoV-2 RNA detection	Yes	78.4 (951)	76.0–80.7
	No	21.6 (262)	19.3–24.0

^a^ Interquartile range

Briefly, their mean age was 60.5 ± 21.5 years and females slightly prevailed (58.3%). As expected, most (78.6%) samples were positive for SARS-CoV-2.

Nineteen out of 1,213 (1.6%) samples resulted positive for RSV, giving an overall prevalence rate of 1.6% (95% CI: 0.9–2.4%). The overall detection rates among working-age (18–64 years) [1.7% (95% CI: 0.9–3.0%)] and older (≥ 65 years) [1.4% (95% CI: 0.6–2.8%)] adults were similar (*p* = 0.83). Most positive cases were detected in December 2021 when the prevalence was as high as 4.6% (95% CI: 2.2–8.3%). In the subsequent three months of 2022 [January: 2.0% (95% CI: 0.8–4.0%); February: 0.5% (95% CI: 0.1–1.9%); March: 0.0% (95% CI: 0.0–1.4%)], the prevalence of RSV gradually decreased.

Twenty-three samples [1.9% (95% CI: 1.2–2.8%)] were positive for influenza viruses and this prevalence was similar to that of RSV (*p* = 0.64). Of influenza positive samples, 91.3% (21/23) and 8.7% (2/23) belonged to A and B types, respectively. Contrary to RSV detections that mostly occurred in December 2021, most (82.6%; 19/23) influenza virus detections were reported in March 2022.

Of specimens tested positive for RSV by the Resp-4-Plex assay, 18 samples proved also positive by the Allplex RP 1. RSV-A (44.4%; 8/18) and RSV-B (55.6%; 10/18) were detected in similar proportions. As shown in Table 2, most (72.2%; 13/18) RSV-positive subjects tested positive for at least one other respiratory pathogen. SARS-CoV-2 (*n* = 7), SP (*n* = 3) and Rhinovirus (*n* = 2) were the most frequent co-detections. Seasonal coronavirus 229E, enterovirus and HI were detected once each. No RSV/ influenza virus co-detections were found.

**Table 2:** Characteristics of RSV-positive subjects

#	Sex	Age, years	Sample date	RSV		Co-detections (Ct)
				Resp-4-Plex (Ct)	Allplex Respiratory Panel 1 (Ct)	
1	F	42	13/12/2021	POS (22.17)	RSV-B (24.79)	SP (27.42)
2	F	86	16/12/2021	POS (22.43)	RSV-A (28.25)	–
3	F	71	16/12/2021	POS (21.65)	RSV-B (23.68)	–
4	M	70	19/12/2021	POS (19.35)	RSV-A (22.56)	–
5	F	29	20/12/2021	POS (18.91)	RSV-A (25.55)	RV (39.09)
6	M	47	20/12/2021	POS (20.47)	RSV-A (23.38)	SP (27.96)
7	F	52	20/12/2021	POS (21.09)	RSV-A (24.94)	SARS-CoV-2 (26.86); HI (38.33)
8	F	30	26/12/2021	POS (18.18)	RSV-A (22.66)	SARS-CoV-2 (23.81); RV (39.33)
9	M	53	26/12/2021	POS (37.31)	RSV-B (41.79)	SARS-CoV-2 (31.90)
10	F	79	28/12/2021	POS (21.39)	RSV-B (25.62)	–
11	F	21	02/01/2022	POS (25.88)	RSV-B (25.09)	CoV-229E (25.42); SP (32.94)
12	F	21	02/01/2022	POS (31.77)	RSV-B (33.39)	EV (33.75)
13	F	50	02/01/2022	POS (23.55)	RSV-B (26.16)	–
14	F	72	03/01/2022	POS (30.57)	RSV-B (33.94)	SARS-CoV-2 (26.47)
15	F	49	03/01/2022	POS (21.65)	RSV-A (29.30)	SP (35.11)
16	F	39	09/01/2022	POS (31.94)	RSV-B (35.31)	SARS-CoV-2 (32.29)
17	M	83	23/01/2022	POS (20.38)	RSV-B (23.13)	–
18	M	91	23/02/2022	POS (34.72)	RSV-A (38.17)	SARS-CoV-2 (30.51)
19	F	91	23/02/2022	POS (36.42)	NEG	SARS-CoV-2 (20.66)

CoV-229E, coronavirus 229E; Ct, cycle threshold; EV, enterovirus; HI, *Hemophilus influenzae*; NA, not available; RV, rhinovirus; SARS-CoV-2, severe acute respiratory syndrome coronavirus 2; SP, *Streptococcus pneumoniae*

### Phylogenetic analysis

**Fig. 1:**
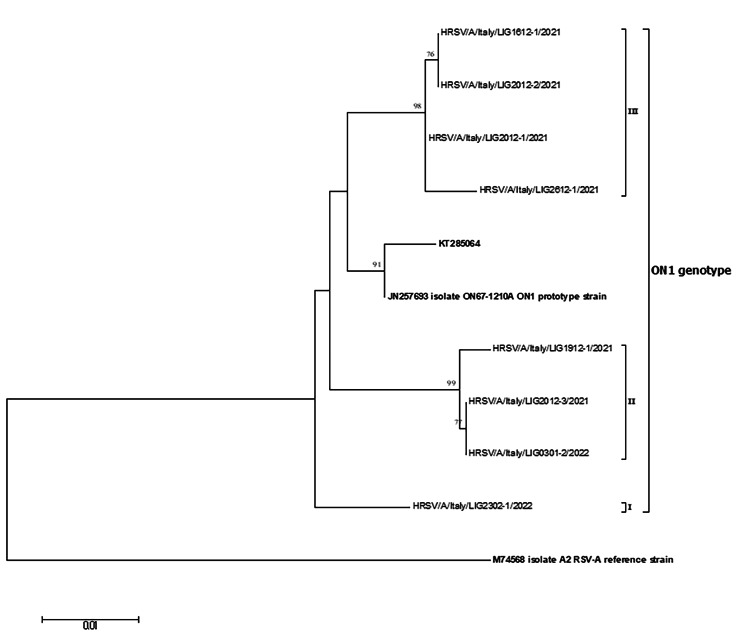
Phylogenetic tree based on the partial G gene sequences of RSV-A strains

Sequencing was successful for 18 samples. As shown in Fig. 1, all RSV-A strains belonged to the ON1 genotype.

Mean nucleotide sequence identity between study RSV-A sequences and the ON1 prototype strain (strain ON67-1210 A, AN: JN257693) was 98.3% (range: 97.9–98.8%). The mean intra-genotypic p-distance of the study ON1 strains was 0.018 ± 0.010. The study sequences were further divided into three branches. In particular, while the HRSV/A/Italy/LIG2302-1/2022 strain was stand-alone (branch I), strains HRSV/A/Italy/LIG1912-1/2021, HRSV/A/Italy/LIG2012-3/2021 and HRSV/A/Italy/LIG0301-2/2022 sequences clustered together (branch II, bootstrap 99%) sharing a mean intra-group p-distance of 0.003 ± 0.002. The remaining four ON1 sequences clustered into the third branch (bootstrap 98%) sharing a mean intra-group p-distance of 0.003 ± 0.003.

**Fig. 2:**
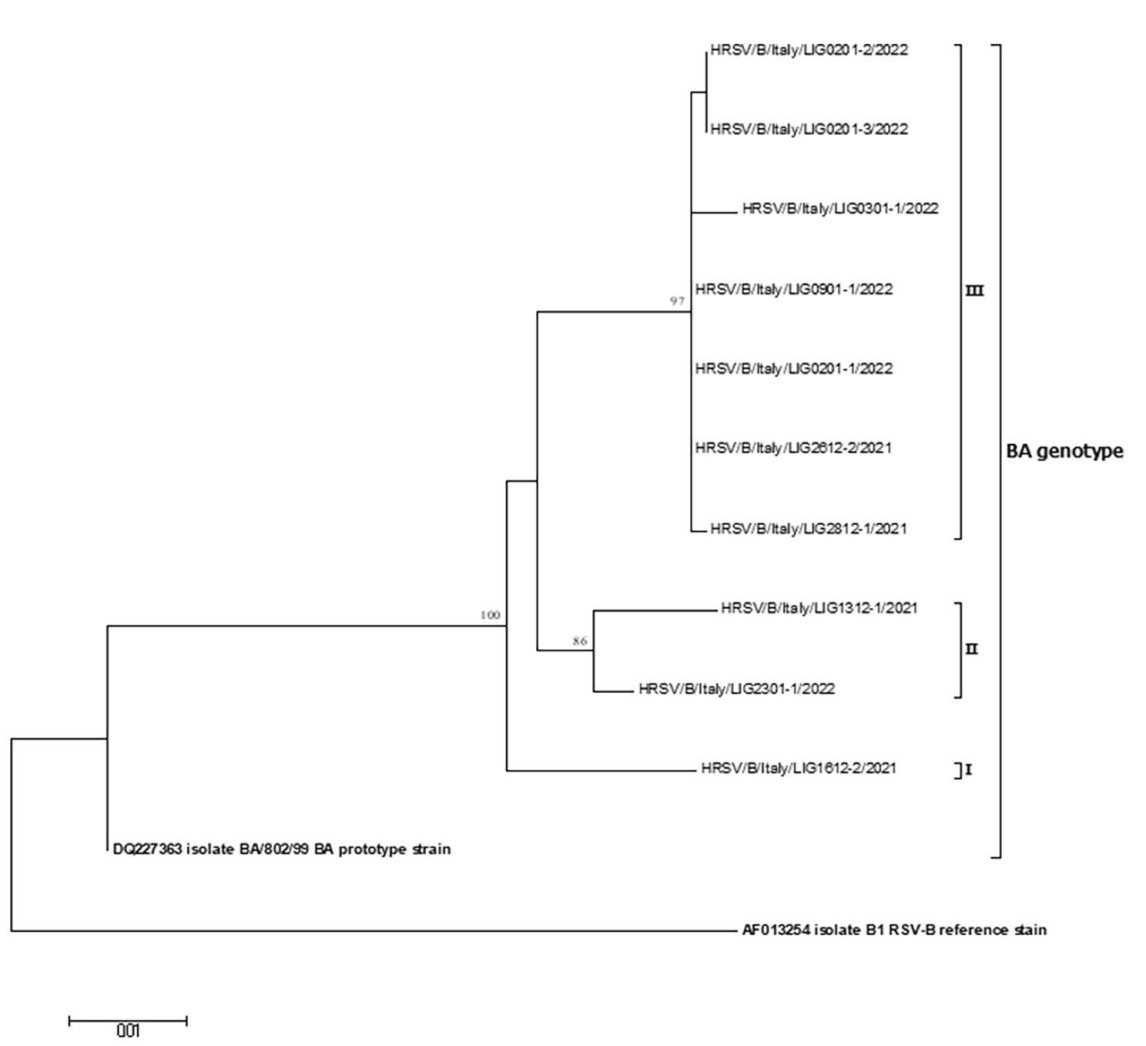
Phylogenetic tree based on the partial G gene sequences of RSV-B strains

For what concerns RSV-B, all study sequences belonged to the BA group and the average nucleotide identity to the BA prototype strain (BA/802/99, AN: DQ227363) was 95.4% (range: 94.9–95.7%). At nucleotide level, the mean intra-genotypic p-distance of the study sequences was 0.014 ± 0.012. The phylogenetic analysis (Fig. 2) showed that the study sequences were further segregated into three branches: strain HRSV/B/Italy/LIG1612-2/2021 was stand-alone (branch I), strains HRSV/B/Italy/LIG1312-1/2021 and HRSV/B/Italy/LIG2301-1/2022 clustered together (branch II, bootstrap 86%) with a mean intra-group p-distance of 0.016, while the remaining seven BA sequences defined branch III (bootstrap 97%) sharing a mean intra-group p-distance of 0.002 ± 0.002.

### Factors associated with RSV infection

In the multivariable analysis, RSV positivity was similar between males and females and between working-age and older adults. On the other hand, the prevalence of RSV among SARS-CoV-2-negative adults was 4.6%, while only 0.7% of SARS-CoV-2-positive subjects were also positive for RSV with an aOR of 5.26 (95% CI: 2.13–14.29).

For what concerns RSV load, subjects with RSV mono-detection (*n* = 6) showed significantly (*p* = 0.016) lower Ct values (mean 21.5 ± 1.5) than those with co-detections (*n* = 13; mean 24.9 ± 2.2) with a |*d*| of 0.93 (95% CI: 0.16–2.02). A similar picture was observed by Allplex RP1 assay (27.0 ± 7.0 vs. 29.9 ± 6.4; *p* = 0.029). This difference was driven by viral co-detections: the Resp-4-Plex Ct values in samples positive for both RSV and other viruses (*n* = 8) were generally lower (mean: 30.0; range: 18.9–37.3) compared with those positive (*n* = 3) for both RSV and bacteria (mean: 21.4; range: 20.5–22.2). For what concerns SARS-CoV-2, subjects negative for RSV (*n* = 944) had on average higher (*p* = 0.095) SARS-CoV-2 load than those with a concomitant (*n* = 7) RSV detection (Ct values of 24.2 ± 8.0 vs. 27.5 ± 7.5).

## Discussion

In the present study, the overall prevalence of RSV in a representative sample of Italian community-dwelling working-age and older adults seeking for SARS-CoV-2 diagnosis was established. Our main findings may be summarized as follows: (i) during the 2021/22 winter season, up to 4.6% of samples tested positive for RSV and RSV detection rate was similar to that of influenza virus; (ii) RSV-A and RSV-B circulated in approximately equal proportions and a substantial level of phylogenetic diversity was observed within each subgroup; (iii) among RSV-positive subjects, the detection of other respiratory viruses and bacteria was frequent; (iv) as shown by significantly lower Ct values, RSV viral load was higher among mono-detections than co-detections.

Few available studies on the confirmed cases of RSV among Italian adults were conducted on a limited number of individuals sourced from different settings. For instance, the cumulative four-season (from 2014/15 to 2017/18) prevalence of RSV among community-dwelling adults with ILI was 4.8%, 9.3% and 12.0% for subjects aged 16–45, 46–65 and > 65 years, respectively [19]. By contrast, in the 2019/20 winter season only 3.0% of adults with ILI tested positive for RSV [36], which approaches our estimate. A high variability of RSV prevalence has been also reported in hospital setting. While Calderaro et al. [20] reported a positivity rate of 22.6% among adult inpatients with respiratory symptoms, the estimates provided by Leli et al. [37] were 2.0% and 7.4% for working-age and older adults, respectively. The heterogeneity in estimates is likely driven by a number of factors, including winter season (i.e., some seasons are characterized by higher RSV activity [38]), study period (e.g., inclusion of summer months decreases the overall prevalence), inclusion criteria and other features of the study population (e.g., different set of symptoms may have different prediction scores). Finally, our study was conducted during the period in which non-pharmaceutical interventions (e.g., mask wearing) were in place that may have had an impact on RSV circulation as well.

We found that RSV-A and RSV-B circulated in similar proportions. An analogous trend was observed during the 2016/17 Italian winter season [19, 39]. Phylogenetically, all RSV-A and RSV-B detections belonged to the ON1 and BA genotypes, respectively; these latter are currently the predominant strains in Italy [19] and Europe [40, 41]. Within both subtypes, the overall p-distance was 1.4–1.8%, which is coherent with previous Italian studies [19, 21]. Indeed, it is known that multiple RSV genotypes may co-circulate during the same season [19, 21, 22], which may denote that local epidemics are caused by a mix of strains seeded from abroad and persistence of local viruses [42]. Continuous monitoring of the molecular dynamics of the local RSV population is warranted in view of the near future prophylactic and therapeutic opportunities.

In our study, the rate of RSV co-detections, most of which were due to SARS-CoV-2, was relatively high. In our opinion, this finding is mostly driven by the fact that the study period (form December 1, 2021 to March 31, 2022) was characterized by the highest SARS-CoV-2 incidence [28]. Analogously, the study population was composed of communing-dwelling adults seeking for SARS-CoV-2 molecular testing. Other than SARS-CoV-2 viral co-detections were significantly less frequent, which is consistent with previous research [20]. With regard to bacteria co-detections, it is likely that these were ascribable to the transient colonization. Indeed, the nasopharyngeal carriage of both SP and HI is common among Italian adults [43, 44]. We then observed that compared with RSV mono-detections, samples tested positive for both RSV and another respiratory pathogen (especially if viral) showed significantly lower viral loads. These findings are in line with a pediatric study conducted during the 2015/16 season: Ct values in RSV single detections were on median 1.5 points lower (25.5 vs. 27.0; *p* = 0.05) than in RSV co-detections [45]. This observation may be explained by a negative viral inference that has been described for RSV co-infections with influenza A virus, rhinovirus and metapneumovirus [46]. A prospective cohort study on the RSV transmission dynamics [47] has brought to light that prior infection with a respiratory virus may lead to either an up-regulation of innate immunity or non-specific cross-reactivity that reduces shedding of a subsequent infection, while presence of RSV co-infections may indicate a low immunity associated with poor viral clearance. Furthermore, RSV detections are less frequent during the periods characterized by high influenza activity and the incidence of RSV may peak 1–2 months earlier [46]. This is consistent with our results when RSV and influenza virus type A circulated in two distinct periods. Analogously, RSV–MPV and RSV-RV co-infections seems to be relatively rare [46]. In the present study no RSV–MPV co-detections were found, while two subjects also positive for RV had viral loads just above the limit of detection (Ct = 39). Less is instead known about a possible inference between RSV and SARS-CoV-2. Bai et al. [48] have demonstrated that RSV had no effect on SARS-CoV-2 infectivity. Although the systematic evidence suggests that co-detections of both viruses are infrequent [16], most available studies were conducted during the period in which several non-pharmaceutical interventions to contain pandemic were in place, which reduced circulation of other common viruses.

This study has some limitations, which may interfere with the interpretation of our findings. The main study drawback was the lack of itemized clinical record data. The study population was composed of adults with respiratory symptoms – either ARI or ILI – and, based on the previous Italian studies [19, 20, 36–38], it is likely that RSV prevalence differs between these two clinical syndromes. Second, the overall RSV prevalence may be underestimated, since most detections occurred in December 2021, while no samples collected earlier were available. In other words, it appears likely that RSV circulated in earlier months of 2021 and therefore we cannot rule out an earlier incidence peak. In this regard, Movva et al. [9] have established that the COVID-19 pandemic may have changed the RSV epidemiology and the number of RSV detections is likely underestimated. Third, the phylogenetic analysis was performed on the G gene only and therefore some important evolutionary patterns in other genome regions were not assessed. Indeed, the lack of whole RSV genome data was identified as a main knowledge gap [49]. Finally, considering the cross-sectional nature of the study, for samples positive for RSV and other respiratory pathogens we were unable to rule out which infection occurred first.

In conclusion, during the 2021/22 winter season, which was characterized by the predominant circulation of SARS-CoV-2 variants of concern Delta and Omicron, a significant proportion of Italian adults were positive for genetically diversified strains of both RSV subgroups. In view of the near future availability of RSV vaccines, the establishment of a national surveillance system should be seen as a public health priority. Future large-scale longitudinal studies should investigate the natural history of an RSV episode (e.g., incidence, complications, prescriptions, outcomes) in adults since these data are essential for policy-oriented health technology assessment of the upcoming vaccines.

## Data Availability

The datasets generated and/or analysed during the current study are not publicly available due to the data nature but are available from the corresponding author on reasonable request.

## List of abbreviations

AdV: Adenovirus
AN: Accession number
aOR: adjusted odds ratio
ARI: Acute respiratory infection
BoV: Bocavirus
BP: Bordetella pertussis
BPP: Bordetella parapertussis
CoV: Coronavirus
CP: Chlamydophila pneumoniae
Ct: Cycle threshold
EV: Enterovirus
HI: Haemophilus influenzae
ILI: Influenza-like illness
LP: Legionella pneumophila
MP: Mycoplasma pneumoniae
MPV: Metapneumovirus
PIV: Parainfluenza virus
RP: Respiratory panel
RSV: Human respiratory syncytial virus
RSV-A: Human respiratory syncytial virus subgroup A
RSV-B: Human respiratory syncytial virus subgroup B
RT-PCR: Real-time reverse transcription polymerase chain reaction
RV: Rhinovirus
SARS-CoV-2: Severe acute respiratory syndrome coronavirus 2
SD: Standard deviation
SP: Streptococcus pneumoniae
TCID: Tissue culture infectious dose
UTM: Universal transport medium
